# The Electromagnetic Vibration Energy Harvesters Utilize Dual-Mass Pendulums for Multidirectional Harvesting

**DOI:** 10.3390/s25072017

**Published:** 2025-03-23

**Authors:** Chong Gao, Xunwen Su, Jiahui Tang, Dongdong Liu, Junlong Liu

**Affiliations:** School of Technology, Beijing Forestry University, Beijing 100080, China; gaochong1999@bjfu.edu.cn (C.G.); jiahui0126@bjfu.edu.cn (J.T.); 15655539873@163.com (D.L.); gc15161157716@163.com (J.L.)

**Keywords:** dual-mass pendulum, vibration energy harvest, frequency tuning, electromagnetic, multidirectional

## Abstract

While vibration harvesting shows promise for powering sensors, effectively harvesting low-frequency, multidirectional ambient vibrations remains challenging. This article presents a novel electromagnetic vibration energy harvesting device (EVEHD) with three key innovations: a dual-mode mass-pendulum configuration—dual-mass coupling (series mode) amplifies induced voltage, and dual-mass uncoupled (parallel) mode enables multifrequency harvesting—spring-position-based frequency tuning (4.5–16.7 Hz in series mode; dual-band 3.7–9.3/5–13.3 Hz in parallel mode), and an optimized energy conversion structure, boosting output by 85.2%. The findings were validated through theoretical modeling, FEM simulations, and shaker tests, the EVEHD generating a maximum voltage of 2 V and a power of 769.2 mW under a base excitation amplitude of 0.5 g at 16.7 Hz. This work reveals the potential of this multidirectional EVEHD for power generation and application in self-powered systems.

## 1. Introduction

With the rapid development of wireless communication, embedded systems, sensor technology, and integrated circuits, microelectronic items generated by these technologies are widely used in many fields such as industry, military, human wearables, biomedical, and environmental monitoring applications [1,2]. The sustainable power supply of microelectronic equipment has become a critical problem. The difficulty of long-term stable power supply of traditional batteries not only limits its application, but also causes environmental pollution due to frequent replacement [3,4]. Therefore, energy harvesting technology has gradually become a feasible solution for microelectronics, utilizing ambient energy sources including solar energy, wind energy, thermal energy, and vibration energy [5,6,7,8,9]. Vibration energy is widely distributed and persistent, and exists in mechanical equipment [10], means of transportation [11,12], and human activities [13,14]. The conversion of vibration energy into electrical energy has a broad application prospect [15]. Vibration energy harvesting devices (VEHDs) can be divided into piezoelectric [16,17], electromagnetic [18,19,20], and electrostatic types [21,22], which are widely used to convert vibration energy into electricity.

The EVEHD exhibits enhanced voltage output and power density compared to alternative transduction mechanisms. The core magnet–coil transduction mechanism converts kinetic energy through time-varying magnetic flux generated by their relative motion, where performance optimization critically depends on maximizing flux variation rate [23]. However, ambient vibrations exhibit low-frequency, multidirectional, and stochastic characteristics that challenge conventional EVEHDs constrained by narrow resonant bandwidths, and this frequency mismatch issue severely degrades energy conversion efficiency. Researchers have implemented vibration amplification [24,25,26], frequency tuning [27,28,29], and the introduction of nonlinearity [30,31] to improve the performance of EVEHDs. Vibration amplification primarily refers to mechanical amplification mechanisms (e.g., lever, rack and crank slide construction) to enhance oscillator displacement. Resonance tuning optimizes electromechanical transduction efficacy by matching the harvester’s natural frequency with ambient excitations for amplitude amplification. Nonlinear techniques can broaden operational-bandwidth multi-stable dynamics to improve the efficiency of EVEHDs [32].

The EVEHD consists of three parts: the vibration receiving structure, the energy conversion structure, and the energy harvesting circuit. The vibration receiving structure can convert environmental vibrations into oscillator vibrations, typically employing resonant spring–mass or pendulum configurations. Scholars have found that the pendulum mechanism has the characteristics of simple structure and high reliability compared to spring–mass structures, and can be applied to low-frequency vibrations [33]. Su et al. developed a frequency-tunable pendulum-based EVEHD that achieved a broad frequency tuning range (7 Hz), overcoming conventional pendulums’ limitations in harvesting low-frequency vibrations, with demonstrated maximum outputs of 1.37 V electromotive force and 521 mW power, though restricted to unidirectional excitation harvesting [34]. Cai et al. proposed a dual-mass pendulum oscillator with position-modulated frequency tuning, enabling ultra-low natural frequency, though its narrow 1.2 Hz operational bandwidth limited effectiveness in mechanical vibration energy harvesting [35]. Surat et al. developed a dual-decoupled-rolling-pendulum energy harvesting system, achieving frequency tuning through geometric parameter adjustment, enabling simultaneous dual-frequency excitation energy harvesting [36]. Chen et al. developed a stacked-axis chaotic-pendulum hybrid energy harvester, achieving high-efficiency low-frequency energy harvesting, albeit with unpredictable chaotic dynamics [37]. Malaji et al. experimentally and numerically investigated a spring-coupled pendulum array energy harvester, demonstrating output power and bandwidth saturation beyond critical pendulum counts [38]. Magnet–coil energy conversion systems have also undergone extensive innovations. The use of high-performance magnetic materials [39], proper arrangement of multiple permanent magnets (PMs) in a single structure, and increasing the number of energy conversion structures have been reported as effective approaches [40,41]. Tang et al. demonstrated a non-resonant EVEHD with enhanced output voltage through magnet configuration and quantity optimization [42]. Halim et al. enhanced radial coil flux and magnetic flux density of coil by introducing soft-iron-embedded cylindrical magnets through magnetization direction and soft-iron size optimization [43]. Li et al. developed a coil/magnet-array EVEHD, analyzing rectangular magnet field distribution, coil flux, and variation rates to identify an optimized magnetic configuration enhancing power density [44]. In summary, critical bottlenecks in EVEHD development are addressable through multiple approaches.

This study proposes a frequency-tunable EVEHD based on a dual-mass pendulum with three distinctive innovations. Firstly, mechanically simplified frequency tuning achieved solely through spring position adjustment eliminates complex auxiliary systems. Secondly, mode-reconfigurable operation enables dual harvesting strategies, with a parallel configuration for multifrequency excitation capture versus series arrangement for voltage amplification via coupled pendulums. Finally, compared to a conventional pendulum, VEHD achieves low-frequency adjustable multidirectional collection. The optimized energy conversion structure improves the output.

The rest of this article is organized as follows. Section 2 presents two topological configurations of the dual-mass pendulum EVEHD, deriving motion differential equations via Lagrange mechanics, complemented by numerical verification of natural frequency characteristics. Section 3 systematically investigates operational bandwidth and power output through electromechanical simulations, optimizing parameters for key components. Section 4 experimentally verifies harvesting performance using shaker-based harmonic excitation. The Section 5 synthesizes the research results.

## 2. Theoretical Study of Dual-Mass EVEHD

**Figure 1:**
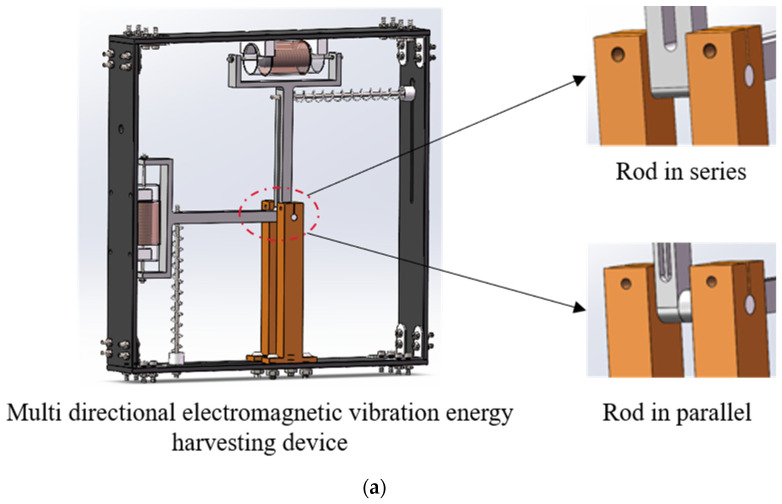

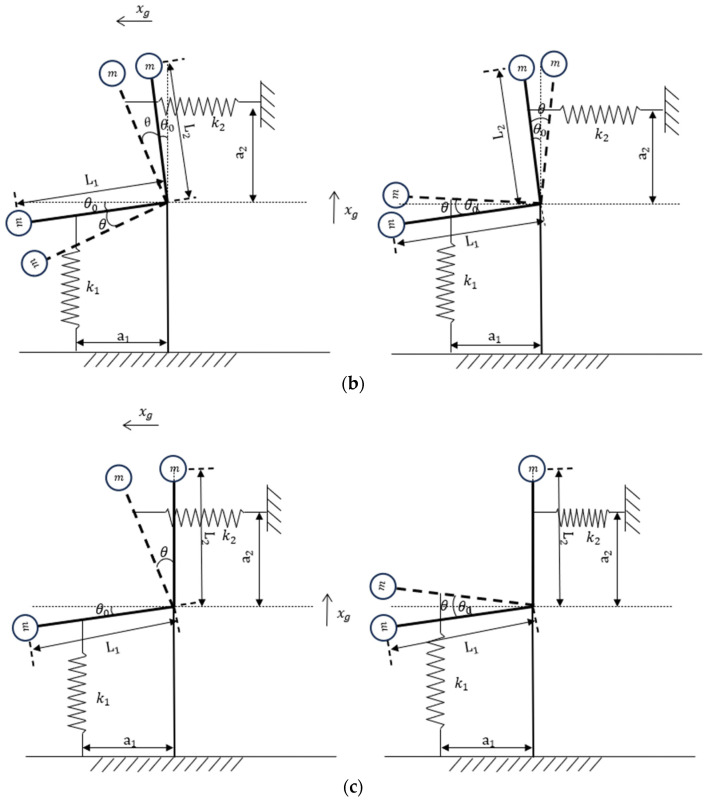
Dual-mass EVEHD configurations: (**a**) 3D schematic diagram; (**b**) series configuration—two pendulums are rigidly connected at the center of rotation; (**c**) parallel configuration—two pendulums are separate and do not affect each other.

Figure 1a illustrates the multidirectional EVEHD configuration, comprising a housing, springs, pendulums, and an energy conversion structure. The pendulums operate in series or parallel configurations via rigid-end connections or separation, with cylindrical-hinged housing connections enabling axial rotation. The energy conversion system employs dual-mass-equivalent PMs mounted on pendulum ends for synchronized rotation and coils fixed to the housing via sleeves surrounding the magnets. Figure 1b presents the series-connected dual-mass EVEHD concept, where a pendulum-linked configuration with housing-mounted springs enables axial oscillation, inducing coil flux variation via magnet motion. The system is subjected to electromagnetic damping and parasitic damping.

As shown in Figure 1b, the system is affected by both horizontal and vertical base motions. Generally, the rotation angle of the pendulum is very small, i.e., sin⁡θ≈θ, cos⁡θ≈1. The differential equation of motion for the multidirectional EVEHD with pendulums connected in series can be obtained through the Lagrange method:

(1) $$\begin{array}{c} \left(L_{1}^{2}+L_{2}^{2}\right)\left(m+\frac{1}{3}m_{1}\right)\ddot{\theta }+\left(c_{t}+c_{p}\right)\left(L_{1}^{2}+L_{2}^{2}\right)\dot{\theta }-\left(mgL_{2}-k_{1}a_{1}^{2}-k_{2}a_{2}^{2}\right)\theta \\ +\left(mgL_{2}-k_{1}a_{1}^{2}-k_{2}a_{2}^{2}\right)\theta_{0}+mgL_{1}\\ =-[\left(mL_{1}+m_{1}L_{1}\right)\cos\left(\theta -\theta_{0}\right)\\ -\left(mL_{2}+m_{1}L_{2}\right)\sin\left(\theta -\theta_{0}\right)]{\ddot{x}}_{g} \end{array}$$

where $L_{1}$ and $L_{2}$ are the lengths from the centroids of the masses to the axis of rotation, respectively; m is the mass of each block; $m_{1}$ is half of the total mass of the pendulum rod; and $c_{t}$ and $c_{p}$ are the electromagnetic damping coefficient and parasitic damping coefficient, respectively. The elastic coefficients of the two springs are $k_{1}$ and $k_{2}$, respectively, and the relative distances between the springs and the rotation axis are $a_{1}$ and $a_{2}$. When the horizontal (vertical) displacement of the base is $x_{g}$, the angle of rotation of the pendulum relative to the initial position is θ, which is the only freedom of the pendulum. The detailed derivation of Equation (1) is given in Appendix A. Equation (1) is transformed into the form of a second-order linear damped spring–mass system:

(2) $$\ddot{\theta }+\frac{C}{M}\dot{\theta }+\frac{K}{M}\theta =A_{0}\sin\left(\theta +\alpha \right){\ddot{x}}_{g}+N$$

(3) $$C=\left(c_{t}+c_{p}\right)\left(L_{1}^{2}+L_{2}^{2}\right)$$

(4) $$M=\left(L_{1}^{2}+L_{2}^{2}\right)\left(m+\frac{1}{3}m_{1}\right)$$

(5) $$K=k_{1}a_{1}^{2}+k_{2}a_{2}^{2}-mgL_{2}$$

(6) $$A_{0}=\sqrt{{\left(mL_{2}+m_{1}L_{2}\right)}^{2}+{\left(mL_{1}+m_{1}L_{1}\right)}^{2}}$$

(7) $$\alpha =\tan^{-1}-\frac{mL_{2}+m_{1}L_{2}}{mL_{1}+m_{1}L_{1}}+\theta_{0}$$

(8) $$N=\left(mgL_{2}-k_{1}a_{1}^{2}-k_{2}a_{2}^{2}\right)\theta_{0}+mgL_{1}$$

In the above equations, C, M, and K are the equivalent damping, mass, and stiffness of the vibration model, respectively. Therefore, the natural frequency of the system can be expressed as:

(9) $$f=\frac{1}{2\pi }\sqrt{\frac{k_{1}a_{1}^{2}+k_{2}a_{2}^{2}-mgL_{2}}{\left(L_{1}^{2}+L_{2}^{2}\right)\left(m+\frac{1}{3}m_{1}\right)}}\;$$

Equation (9) demonstrates tunable-system natural frequency through $a_{1}$ and $a_{2}$ modulation. However, the natural frequency is also related to $L_{1}$, $L_{2}$, $k_{1}$, $k_{2}$, m, and $m_{1}$, and these parameters remain fixed post-fabrication and are treated as system constants during operation. In addition, when $k_{1}a_{1}^{2}+k_{2}a_{2}^{2}\gg mgL_{2}$, $k_{1}=k_{2}$, the natural frequency f can be regarded as a linear function with the independent variable a and the slope of $\sqrt{k_{1}+k_{2}}$. The solution to Equation (2) combines the general solution $\theta_{1}\left(t\right)$ and particular solution $\theta_{2}(t)$ of a constant-coefficient linear homogeneous second-order differential equation:

(10) $$\theta \left(t\right)=\theta_{1}\left(t\right)+\theta_{2}(t)$$

In the formula, $\theta_{1}(t)$ represents the free vibration of a damped system, and the damping is usually relatively small. Therefore, $\theta_{1}\left(t\right)$ can be expressed as:

(11) $$\theta_{1}\left(t\right)=Ae^{-\tau t}\sin\left(\omega_{1}+\varphi_{1}\right)$$

The vibration amplitude exponentially decays to zero, existing only transiently post-initiation, thus requiring analysis of only the steady-state solution, permitting omission of the solution $\theta_{1}\left(t\right)$.

$\theta_{2}(t)$ is the steady-state solution of the system, representing the forced vibration of a damped system. It is assumed that the input excitation is sinusoidal, and therefore the expression of the specific solution contains a sine function. The input excitation can be set to:

(12) $$F=F_{0}\sin\omega t=M_{m}{\ddot{x}}_{g}\;$$

The specific solution is:

(13) $$\theta_{2}\left(t\right)=B\sin\left(\omega t-\varphi \right)+MN/K$$

(14) $$B=\frac{A_{0}F_{0}}{M_{m}\sqrt{{\left(\frac{K}{M}-\omega^{2}\right)}^{2}+{\left(\frac{C\omega }{M}\right)}^{2}}}$$

(15) $$\varphi =arc\tan\frac{C\omega }{K-M\omega^{2}}\;$$

where B is the amplitude of the pendulum, φ is the phase angle, and $M_{m}$ is the total mass of the system. From Equations (13)–(15), it can be seen that, when the device is subjected to sinusoidal excitation, the forced vibration of the damped system is also a sinusoidal vibration with frequency equal to the excitation frequency ω. The amplitude B and phase angle φ are related to the magnitude and frequency of the excitation force, the total mass, the elastic coefficients of springs, the mass of the block, the damping coefficients, etc., and are independent of the initial conditions. When $\omega =\omega_{n}$, the amplitude is maximum, so the natural frequency of the VEHD should be close to the frequency of the input excitation force.

Figure 1c presents the parallel-connected dual-mass conceptual schematic, featuring decoupled pendulum termini with kinematically independent motions. The vertical pendulum exhibits zero theoretical initial angle. Motion equations can be derived via Lagrange’s method:

(16) $$\left(mL_{1}^{2}+\frac{1}{3}m_{1}L_{1}^{2}\right)\ddot{\theta }+k_{1}a_{1}^{2}\theta +\left(c_{t}+c_{p}\right)L_{1}^{2}\dot{\theta }=-mL_{1}{\ddot{x}}_{g}+mgL_{1}\;$$

(17) $$\left(mL_{2}^{2}+\frac{1}{3}m_{1}L_{2}^{2}\right)\ddot{\theta }+k_{2}a_{2}^{2}\theta +\left(c_{t}+c_{p}\right)L_{2}^{2}\dot{\theta }=-mL_{2}{\ddot{x}}_{g}\;$$

The meaning of each symbol is the same as above. The derivation process of Equations (16) and (17) is similar to that of Equation (1). The natural frequency of the two systems can be obtained through Equations (16) and (17), as follows:

(18) $$f_{1}=\frac{1}{2\pi }\sqrt{\frac{k_{1}a_{1}^{2}}{\left(m+\frac{1}{3}m_{1}\right)L_{1}^{2}}}\;$$

(19) $$f_{2}=\frac{1}{2\pi }\sqrt{\frac{k_{2}a_{2}^{2}}{\left(m+\frac{1}{3}m_{1}\right)L_{2}^{2}}}\;$$

Equations (18) and (19) reveal linear natural frequency tuning in the parallel device through spring position modulation. Moreover, different spring sizing enables dual-frequency tuning ranges. Near-rotation-center spring placement can achieve ultra-low theoretical frequencies, and address conventional pendulum limitations.

**Figure 2:**
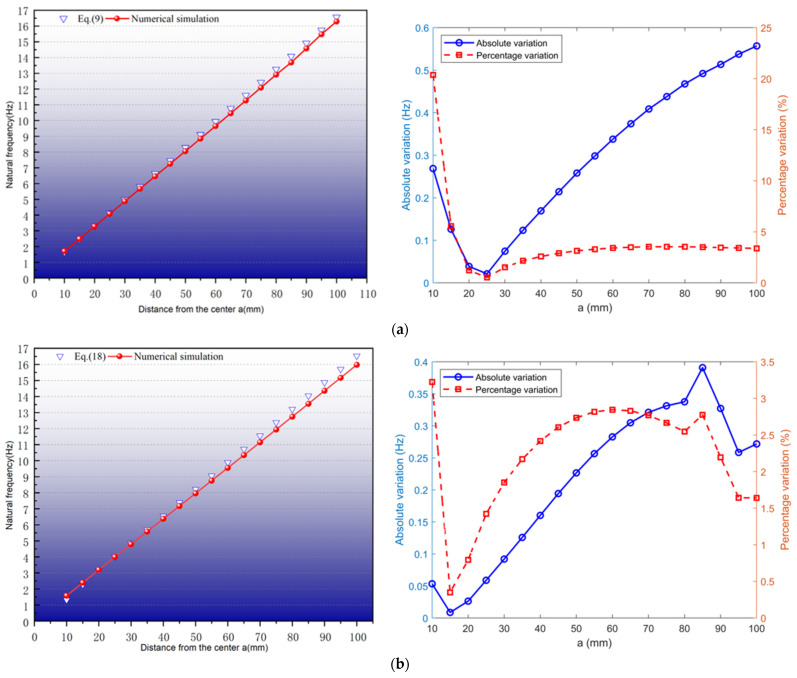
Theoretical and simulation results and error analysis. (**a**) Series; (**b**) parallel.

A simplified device model was constructed, with the key parameters detailed in Table 1. Theoretical derivations were numerically verified through modal simulations. The computational complexity is reduced by replacing the PM with a block of equal mass and omitting the energy conversion structure. Consistency with the theoretical model is maintained. The data in Table 1 were substituted into Equation (9) and Equation (18), respectively. The parameter a was incrementally adjusted from 10 mm to 100 mm in 5 mm steps under condition $a_{1}=a_{2}$. The results and error analysis of the numerical simulation and theoretical calculations are given in Figure 2.

As can be seen in Figure 2, the theoretical and simulated natural frequencies exhibit close agreement. The coefficient of determination ($R^{2}=0.99$) confirms strong correlation between theoretical predictions and FEM simulations. We find a mean deviation of 0.303 Hz and peak discrepancy of 0.557 Hz (at a = 100 mm) for series configuration, while a mean deviation of 0.212 Hz and peak discrepancy of 0.39 Hz (at a = 85 mm) for parallel configuration. Moreover, Figure 2 shows the theoretical and simulated absolute and percentage error curves for both systems. In addition, when ‘a’ is synchronously adjusted, the natural frequency of the system varies linearly, which confirms the accuracy of the proposed theoretical model. The theoretical slope is greater than the simulation slope. This discrepancy is primarily attributed to computational errors in determining the elastic coefficient of springs.

This paper investigated the performance of the proposed multidirectional EVEHD. Taking the double-mass pendulum in series as an example, the average power dissipated by the damping element is:

(20) $$P_{c}=\frac{1}{T}\int_{0}^{T}c\dot{\theta }\left(t\right)\cdot \dot{\theta }\left(t\right)dt=\frac{CB^{2}\omega^{2}}{2(L_{1}^{2}+L_{2}^{2})}=\frac{MA_{0}^{2}F_{0}^{2}\zeta z^{2}}{M_{m}^{2}\omega_{n}\left(L_{1}^{2}+L_{2}^{2}\right)\;[{\left(1-z^{2}\right)}^{2}+{\left(2\zeta z\right)}^{2}]}$$

**Figure 3:**
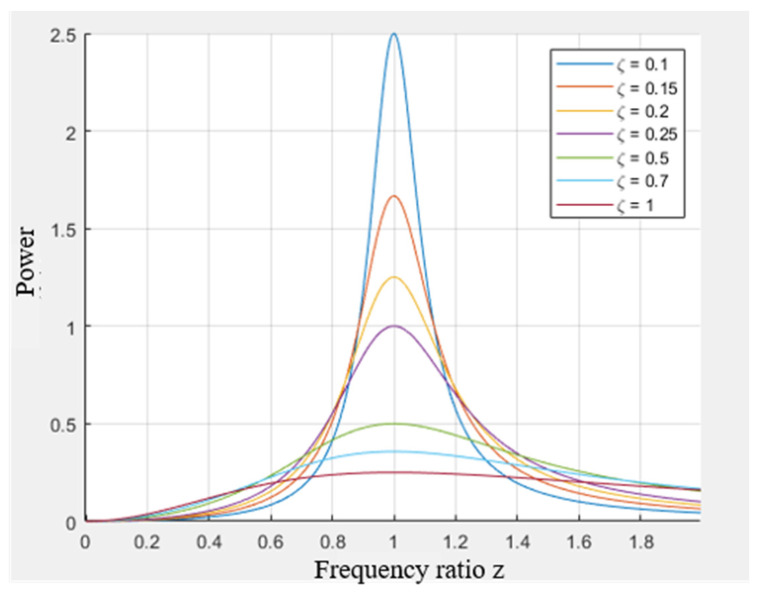
Damping element power curve.

In the formula, $\frac{\omega }{\omega_{n}}=z$, which is called the frequency ratio. $\frac{C}{2M\omega_{n}}=\zeta$, which is called the damping ratio. As illustrated in Figure 3, the damped dissipated average power, calculated in accordance with Equation (20), is presented as a function of the frequency ratio and the damping ratio.

Figure 3 demonstrates average power peaks at unity frequency ratio, confirming resonant synchronization between natural and ambient frequencies. Furthermore, lower damping ratios yield higher maximum power outputs. Assuming that the initial displacement and initial velocity of the base are both 0, $F_{0}/M_{m}=X_{0}\omega^{2}$. For this EVEHD, the final generation of electrical energy is actually negative work generated by electromagnetic force, and its maximum power is:

(21) $$P_{t}=\frac{c_{t}}{c_{t}+c_{p}}P_{c-max}=\frac{c_{t}}{{\left(c_{t}+c_{p}\right)}^{2}}*\frac{{\left(MA_{0}X_{0}\omega_{n}^{2}\right)}^{2}}{2{\left(L_{1}^{2}+L_{2}^{2}\right)}^{2}}\;$$

The Equation (21) takes the maximum value when $c_{t}=c_{p}$, and its expression is:

(22) $$P_{t-max}=\frac{{\left(MA_{0}X_{0}\omega_{n}^{2}\right)}^{2}}{8c_{t}{\left(L_{1}^{2}+L_{2}^{2}\right)}^{2}}$$

From Equation (22), it can be seen that the power of the EVEHD is related to the natural frequency of the device, as well as parameters such as amplitude of excitation, the size of the device, and electromagnetic damping. In addition, load is another key factor affecting power output. According to Faraday’s law of electromagnetic induction, the magnitude of the induced electromotive force in the circuit is proportional to the rate of change of the magnetic flux over time, and it is also proportional to the velocity, with the sign opposite to the velocity. Therefore, $U_{0}$ can be expressed as:

(23) $$U_{0}=-N\frac{d\varphi }{dt}=-\frac{d\psi }{dt}=-\frac{d\psi }{d\theta }\frac{d\theta }{dt}=-\frac{d\psi }{d\theta }\cdot \dot{\theta }\left(t\right)=K_{e}\dot{\theta }(t)$$

where N is the number of turns of the coil, φ is the magnetic flux, and ψ is the total magnetic flux. $K_{e}$ is the back electromotive force constant, which is related to the geometric parameters and magnetism of the electromagnetic damper. Generally, the relative amplitude of the oscillator is small, and the back electromotive force constant is considered to remain constant [45].

According to Lorentz’s law, the electromagnetic damping force applied to a PM is proportional to the current. Due to the motion mode of the PM, the electromagnetic damping force is represented by the torque $T_{t}$:

(24) $$T_{t}=K_{t}i_{load}\;$$

where $K_{t}$ is the motor constant of the rotating electromagnetic device. Typically, $K_{e}$ and $K_{e}$ can be treated as equal. Therefore, the instantaneous power of the electromagnetic damping can be expressed as:

(25) $$P_{t-I}=T_{t}\cdot \dot{\theta }\left(t\right)=U_{0}i_{load}=c_{t}{\dot{\theta }}^{2}(t)\;$$

(26) $$c_{t}=\frac{K_{e}^{2}}{R_{coil}+R_{load}}\;$$

In summary, the system demonstrates suitability for low-frequency vibration energy harvesting, though achieving optimal performance requires strategic optimization of structural parameters (e.g., device dimensions, magnet–coil configuration), and subsequent analyses will focus on maximizing efficiency.

## 3. Simulation and Optimization

The dual-mass EVEHD’s performance is governed by energy conversion and pick-up structures, with parametric influences systematically evaluated through modal and electromagnetic simulations. Bidirectional harvesting capability was validated through dynamic analysis of the pick-up structure.

**Figure 4:**
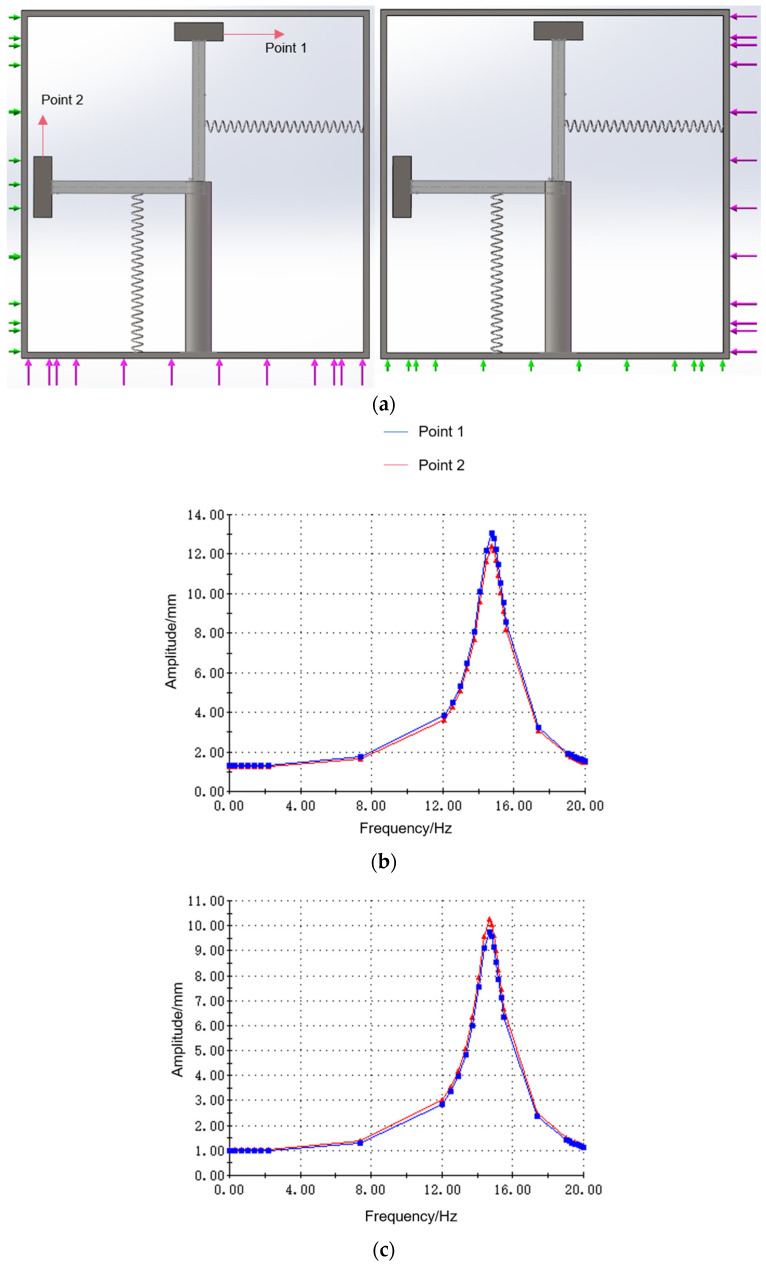
Device subjected to bidirectional excitation (0–20 Hz, 0.5 g). (**a**) Schematic diagram; (**b**) frequency response under vertical excitation; (**c**) frequency response under horizontal excitation.

First, the amplitude response of the system when subjected to X/Y excitation is investigated. The system’s natural frequency is experimentally determined as 15.2 Hz when springs are positioned at $L_{1}=L_{2}=45\;mm$ (see Figure 4a). Horizontal and vertical excitations are applied to the device, respectively. Purple arrows indicate the excitation, and green arrows indicate that the device is constrained to move in this plane. Points 1 and 2 were selected on the two masses to study the response amplitude. The excitation is swept from 0 to 20 Hz with a magnitude of 0.5 g and a direction perpendicular to the shell surface. The response amplitude at points 1 and 2 when the device is subjected to vertical and horizontal excitation, respectively, is shown in Figure 4b,c. Frequency sweeps (0–20 Hz) revealed resonant peaks at 15.2 Hz in both directions, demonstrating bidirectional harvesting capability. Mass response amplitudes at points 1 and 2 exhibited minor variance (<5%) due to error generated when selecting the point location.

Key pick-up structure parameters—magnet mass, pendulum length, and spring stiffness—govern frequency tuning performance. Real-time spring positioning enables operational frequency tuning, while pre-production parameter optimization allows scenario-specific frequency ranges, with differential parametric impacts on tuning efficacy necessitating further investigation.

**Figure 5:**
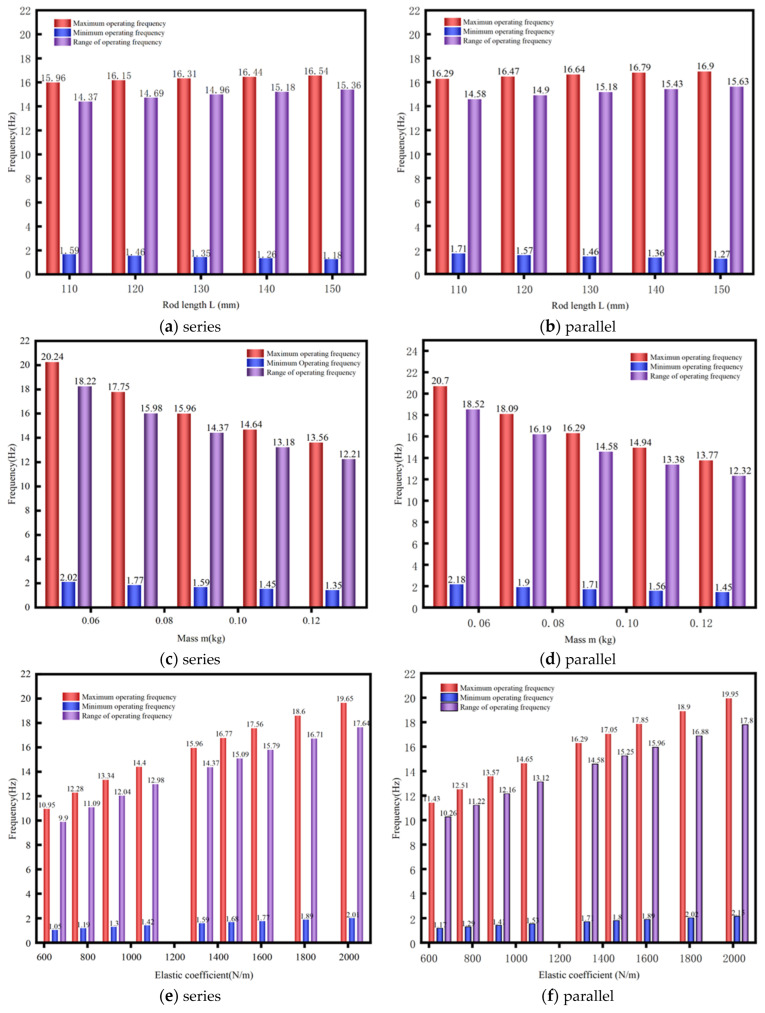
Relationship between operating frequency and structural parameters.

Figure 5 characterizes parametric influences on the multidirectional EVEHD’s frequency tuning range, with Figure 5a,b specifically illustrating pendulum length versus frequency tuning range.

Pendulum lengthening induces minimal natural frequency variation (both $f_{min}$ and $f_{max}$), with extended rod dimensions marginally improving spring adjustability but disproportionately increasing system volume, which is counterproductive to miniaturization. The pendulum length was therefore constrained to ≤150 mm from the rotation center to prioritize compact design.

Figure 5c,d show the relationship between the natural frequency tuning range and the mass of the PM. When the mass of the PM is excessive, this leads to an increase in the overall mass of the device, and when the mass of the PM is excessively small, this leads to a reduction in the number of magnets and in the amplitude of energy harvesting efficiency. A PM mass of the device in the frequency range of 0.06–0.1 kg is optimal. The PM mass was precisely quantified through electromagnetic simulation optimization.

Figure 5e,f show the relationship between the natural frequency and the spring’s elastic coefficient. Comparative analysis of Figure 5 reveals that elastic coefficient exerts the dominant influence on natural frequency, with coefficient enhancement substantially expanding the frequency tuning range. The evaluated elastic coefficient attenuates pendulum oscillations, compromising coil flux variation rates and adversely affecting power conversion. The amplitude in Figure 4 is reasonable, so the spring has been designed to keep the elastic coefficient similar to the initial parameter. The above analysis provides a reference for the final optimization of the actual device size.

**Figure A1:**
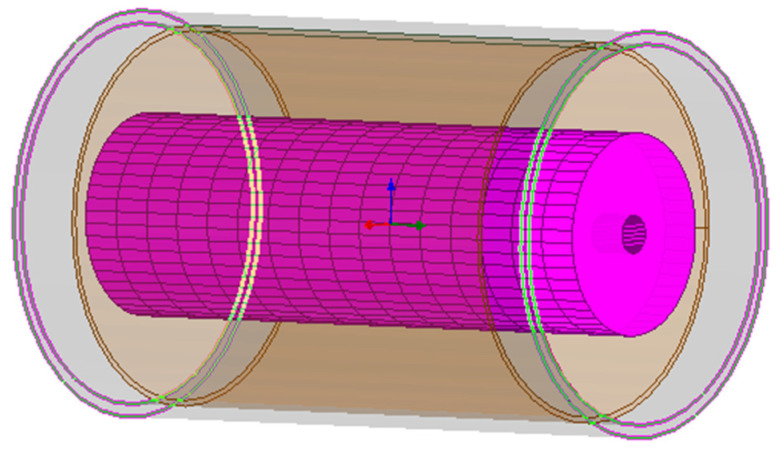
Simulation model.

The performance of the energy conversion structure is the crucial factor for the energy efficiency of the whole device. In this paper, an energy conversion structure combining PMs, coils, and soft magnetic materials is proposed to improve its working performance. Electromagnetic simulation is used to optimize the proposed structure. The simulation includes the static simulation of the magnetic flux density in the coil when the PM is stationary and the dynamic simulation of the magnitude of the induced electromotive force in the coil when the PM is moving. The simplified simulation model is displayed in Figure A1. The model shows the soft magnetic shell, the coil, and the PM from the outside inwards. The function of the soft magnetic shell is to allow the magnetic flux to form a closed loop within the coil, reducing magnetic leakage and energy loss [46]. NdFeB permanent magnets were selected for their high magnetic energy density and compact geometry, optimal for electromagnetic energy harvesting applications. Considering cost and accessibility, round PMs with holes were chosen. Multi-magnet arrays enable enhanced flux gradients through strategic geometric arrangements, constituting a critical performance enhancement strategy, hence the use of multiple PM sheets instead of a single PM [44].

Static simulations assessed the effects of geometric parameters (arrangement, size, quantity) of PMs and PM–coil air-gap width on magnetic flux density B. The initial values of each parameter are shown in Table 2.

**Figure 6:**
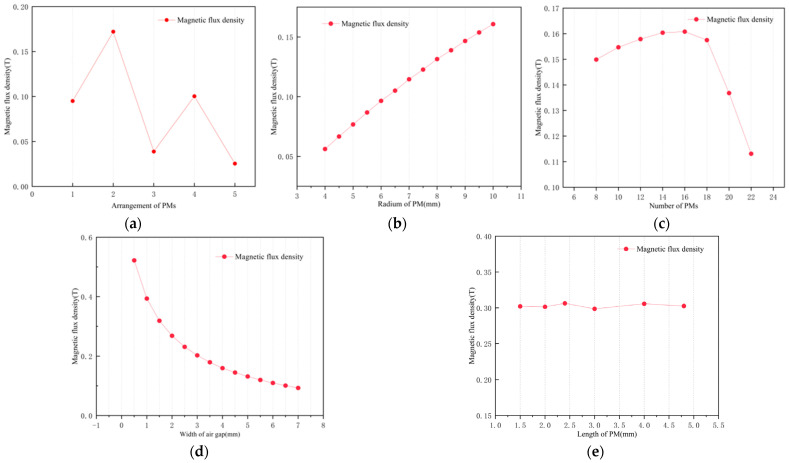
The influence of PM parameters on the magnetic flux density in coil: (**a**) arrangement; (**b**) radius; (**c**) number; (**d**) width of air gap; (**e**) length.

Firstly, the magnetic flux density generated in the coil by several arrays of PMs was investigated. Figure 6a compares five permanent arrangements without a soft magnetic shell: parallel arrangement, the right half arranged in reverse, every two magnets placed in opposite directions, every four magnets placed in opposite directions, and a Halbach array. Maximum magnetic flux density occurs when the right half of the PMs are reversed. The Halbach array concentrates the magnetic field on one side of the array rather than in the coil, but the maximum magnetic field generated by the PM is mostly outside the coil coverage during motion, so this arrangement is clearly unsuitable for the PM–coil structure in this paper.

Setting the right half of PMs arranged in reverse, and adding a soft magnetic shell made of iron outside the coil, the magnetic induction strength in the coil is increased by 0.4 T to 0.2280 T, indicating the superiority of this structure.

Figure 6b–e delineate the parametric sensitivity of PM radius, quantity, and air-gap dimensions to coil flux density. It can be concluded that the coil flux density is positively correlated with the radii of the PMs. If the number of PMs is too large, the coil flux density will decrease. Increasing the air gap width will reduce the coil flux density, but the magnitude of the reduction will also decrease. The length of the PMs has no significant effect on the coil flux density. To summarize, we selected easily obtainable PMs with a length of 3 mm, a quantity of 16, and a radius of 8 mm. The total mass of the PMs can be calculated to be 0.07 kg, which is within a reasonable mass range according to the previous section.

**Figure 7:**
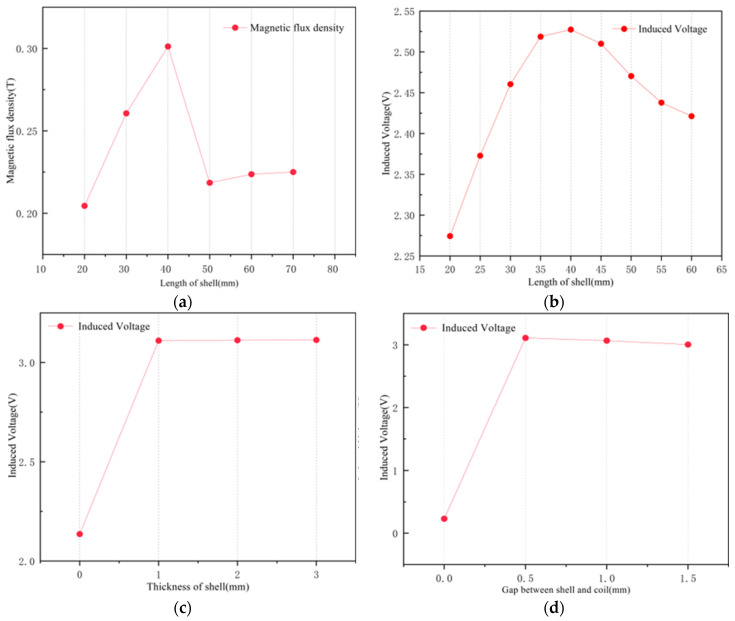
Influence of soft magnetic shell parameters on coil flux density and induced voltage: (**a**) length versus coil flux density; (**b**) length versus induced voltage; (**c**) thickness versus induced voltage; (**d**) gap between shell and coil versus induced voltage.

**Figure A2:**
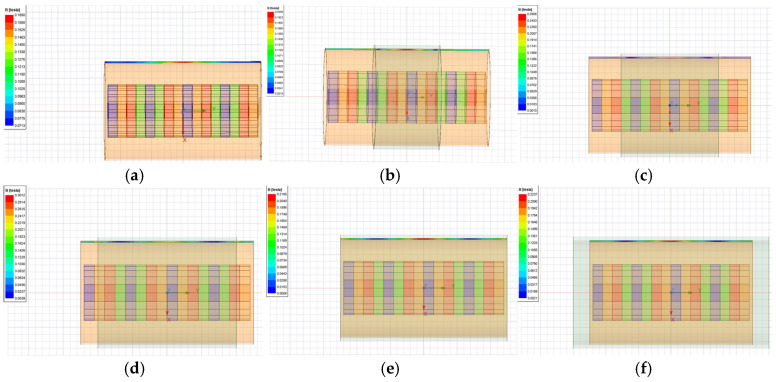
Magnetic field distribution in coils with different widths of soft magnetic materials: (**a**) without soft magnetic materials; (**b**) 20 mm; (**c**) 30 mm; (**d**) 40 mm; (**e**) 50 mm; (**f**) 60 mm.

Figure 7a analyzes the parametric influence of soft magnetic shell thickness on coil flux density, revealing that when the width of the shell is less than the width of the coil, the coil flux density increases with the growth of width. Figure A2 shows the magnetic field distribution in the coil for different widths of the soft magnetic shell, demonstrating that after the increase of the shell, the location of the strongest magnetic flux density in the coil is shifted from the middle to the ends of the iron shell, so that when the length of the iron shell exceeds the width of the coil, there is a huge drop in the magnetic flux density in the coil. Certain parameters of the soft magnetic shell are determined in relation to dynamic simulations.

**Figure 8:**
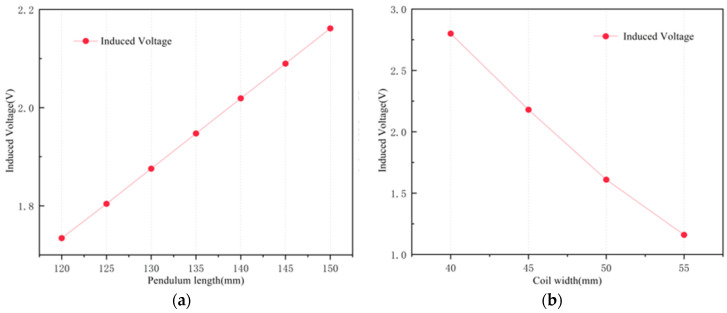
(**a**) The relationship between the pendulum length and induced voltage; (**b**) the relationship between the coil width and induced voltage.

**Figure A3:**
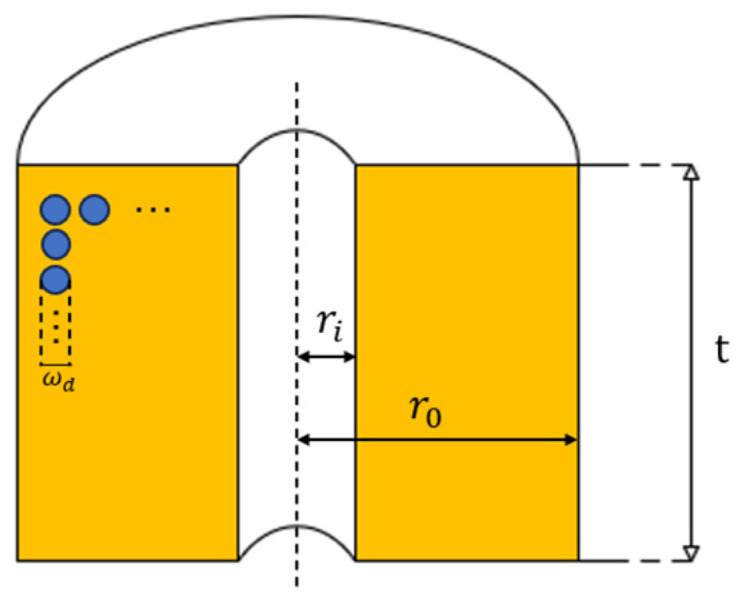
Cross-sectional view of coil.

The parameters of the PMs were optimized using static magnetic field simulations, based on which transient electromagnetic analyses were carried out when the PMs were subjected to harmonic excitation. When performing dynamic simulations, it is crucial to set up the correct equations of motion for the PMs. According to the harmonic analysis in Section 2, the system is subjected to a sine excitation force with an amplitude of 0.5 g and a frequency set at 16 Hz. By changing the arrangement of the PMs, it has been verified that the induced electromotive force in the coil is maximum when the right half of the PMs are reversed. Figure 8a demonstrates the relationship between pendulum length and induced voltage magnitude; it can be seen that the induced voltage is positively correlated with the length of the pendulum, and the moderate length of the pendulum is chosen to be 145 mm. Figure 8b analyzes the effect of coil width on the induced voltage and shows the inverse relationship between them. If the coil width is too narrow, it will result in too great a thickness of the coil winding, which is not conducive to manufacture; therefore, a coil width of 45 mm was adopted. Different wire sizes also affect the value of the induced voltage. A cross-section of the coil winding is illustrated in Figure A3. Coils were fabricated via enameled wire. The fill factor of the copper in the coil depends on the tightness of the winding, the thickness of the insulating sleeve, and the shape of the winding, but winding errors cannot be completely eliminated, and the position of the copper wire cannot be precisely controlled. Using different winding methods, the fill factor can be as high as 90%. Assuming that the copper fill factor of the coil is $K_{coil}$ and the total cross-sectional area of the coil is $A_{coil}$, the resistance of the coil can be calculated using the following equation:

(27) $$R_{coil}=\rho \frac{L_{w}}{A_{w}}=\rho \frac{NL_{MT}}{A_{w}}=\rho \frac{N^{2}\pi (r_{0}+r_{i})}{K_{coil}\left(r_{0}-r_{i}\right)t}\;$$

where $r_{i}$ is the inner radius of the coil, $r_{0}$ is the outer radius of the coil, t is the width of the coil, $\omega_{d}$ is the wire diameter, $A_{w}$ is the area of the conductor, $L_{w}$ is the length of the conductor, $L_{MT}$ is the average length of the wire turns, N is the number of turns of the coil, ρ is the coil resistivity of the material, and $R_{coil}$ is the coil resistance.

We set the number of turns of the coil to 300, simulated and analyzed the induced electromotive force generated by different specifications of the coil, and obtained its resistance value from Equation (27) and calculated the corresponding power; the results are shown in Table A1. Increasing the diameter of the enameled wire will increase the output electric power, but excessive winding thickness will increase the coil flux density gap between the inner and outer coils, so the enameled wire diameter of 0.45 mm is selected and the winding thickness is 1.35 mm.

The relationship between the parameters of the soft magnetic shell and the induced voltage in the coil was simulated and analyzed, including the thickness of the soft magnetic material, the gap between the coil windings and the soft magnetic shell, and the width of the soft magnetic shell. The results are given in Figure 7b–d. Figure 7b shows that the thickness of the soft magnetic shell has little effect on the induced voltage. Analyzing Figure 7d, it can be concluded that the contact between the soft magnetic shell and the coil leads to a sharp decrease in the induced voltage in the coil, and therefore a small gap is required between the soft magnetic material and the coil. The width of the gap does not have a significant effect on the induced voltage. Figure 7c shows the variation of the induced voltage in the coil with changing width of the soft magnetic shell. When the width of the soft magnetic shell is smaller than the width of the coil, increasing the width of the soft magnetic shell increases the voltage value, while when the width of the soft magnetic shell is larger than or equal to the width of the coil, increasing the width of the soft magnetic shell slightly decreases the voltage value. The final parameters of the soft magnetic shell were determined to be 1 mm thick and 40 mm wide, with a 0.5 mm gap to the coil.

## 4. Experiment Validation

**Figure A4:**
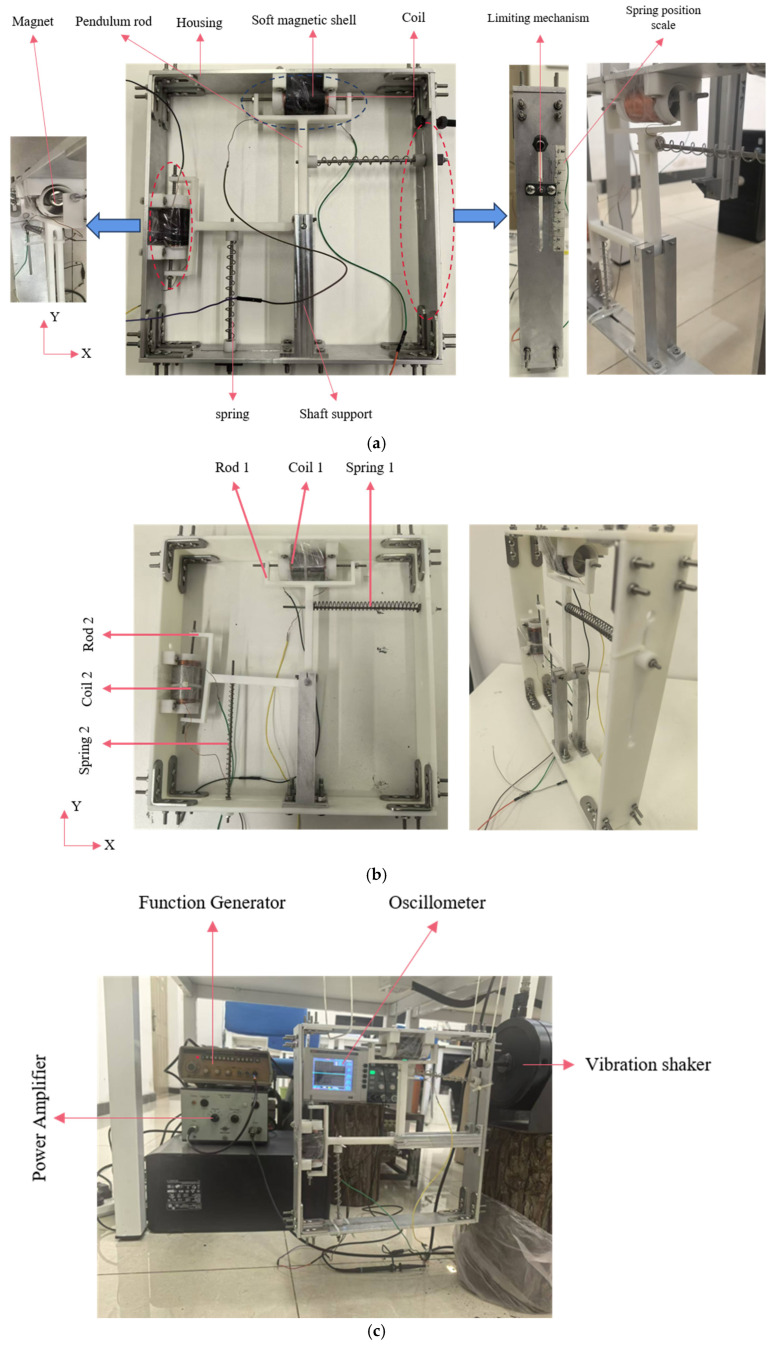

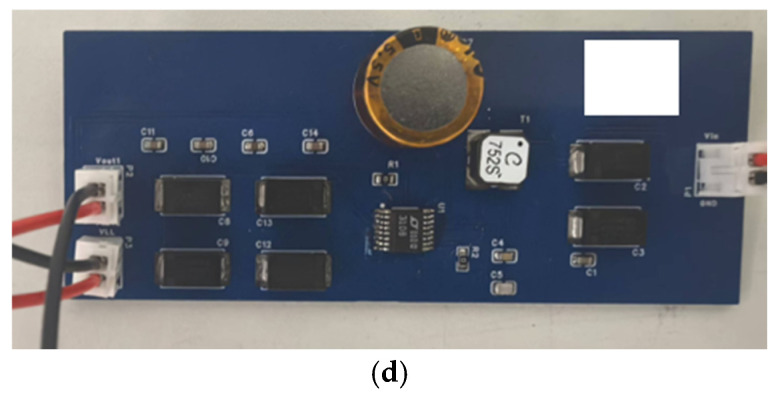
(**a**) Series device; (**b**) parallel device; (**c**) vibration test platform; (**d**) power extraction circuit.

Based on the results of the simulation optimization, the dual-mass EVEHD was fabricated as shown in Figure A4a,b, where XY represents the two directions of excitation inputs. The case materials are aluminum alloy and 3D-printed materials, respectively. The masses of the series and parallel devices are 1.08 kg and 0.45 kg, respectively. It is worthy of mention that the parallel unit is equipped with two distinct types of springs, yet employs the same pendulum and PMs. As shown in Figure A4c, the fabricated device was subjected to a shaker test to evaluate its energy harvesting performance. The shaker generates sine excitation forces with an amplitude of 0.5 g at different frequencies. In the excitation test, a function signal generator (type: GFG-8015G) is connected to a power amplifier (type: Brüel & Kjær2706) and then drives the shaker (type: Brüel & Kjær4809), which is connected to the instrument housing; the instrument is suspended by a thin rope, and the voltage signals generated by the coil and the energy harvesting circuit are recorded by an oscilloscope (type: UT2062CE). To measure the device’s power output, the coil resistance was determined using a multimeter. Figure A4d illustrates an energy harvesting circuit module comprising the LTC3108 chip with peripheral circuits. This circuitry is capable of continuously outputting a 3.3 V DC voltage to the load circuit and storing the collected power in a supercapacitor element.

**Figure 9:**
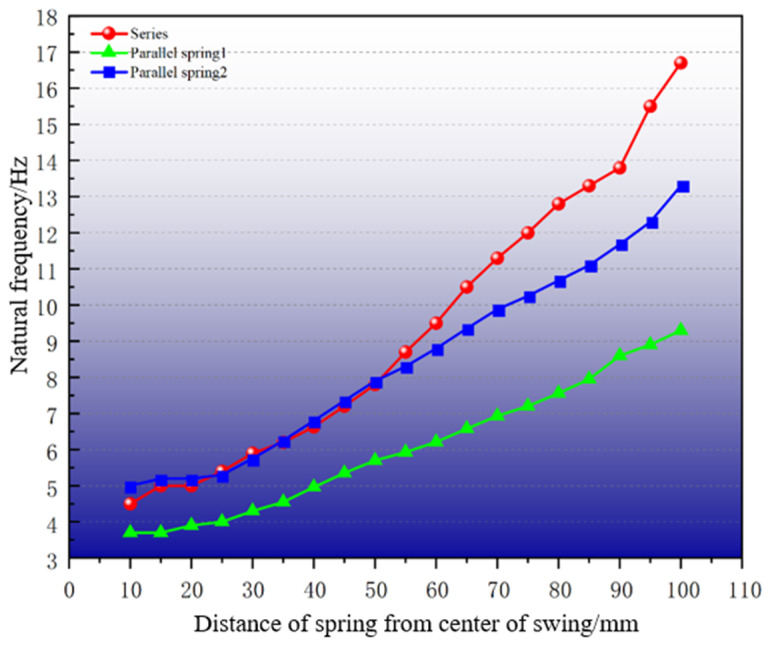
Experimental measurement of the natural frequency tuning range of the series device and parallel device.

**Figure A5:**
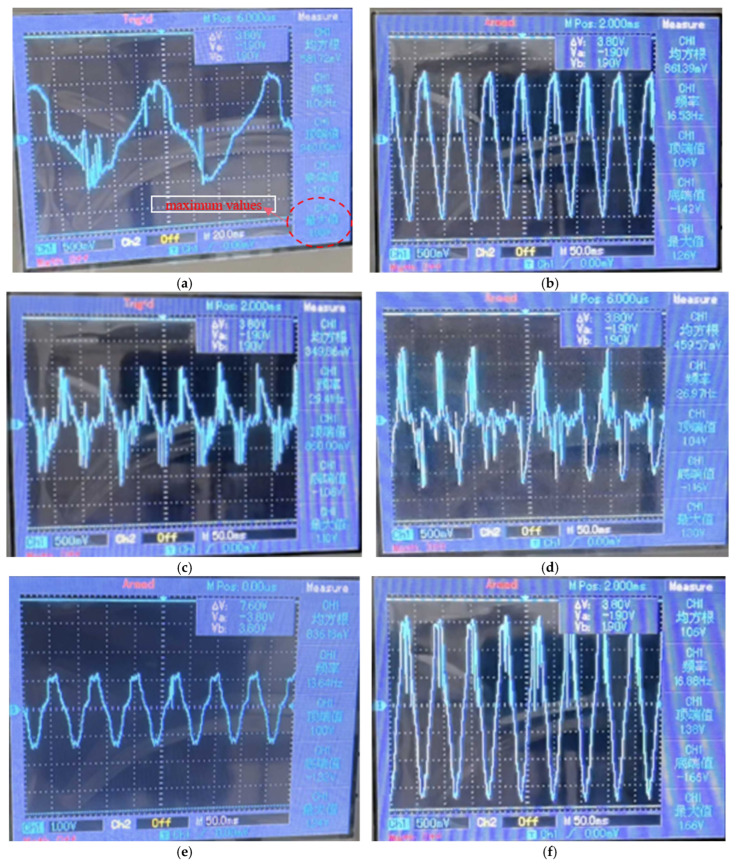

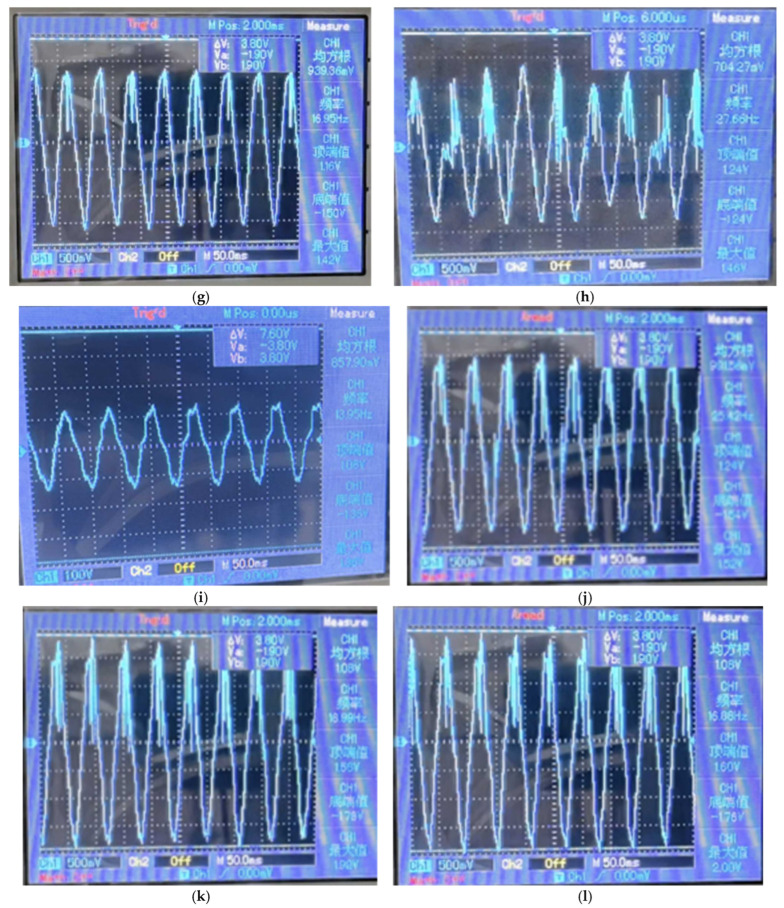
Experimental results of device in series mode. Input 10 Hz excitation: (**a**) without soft magnetic shell (X); (**b**) without soft magnetic shell (Y); (**c**) composite soft magnetic material shell (X); (**d**) composite soft magnetic material shell (Y); (**e**) iron shell (X); (**f**) iron shell (Y). Input 16.7 Hz excitation: (**g**) without soft magnetic shell (X); (**h**) without soft magnetic shell (Y) (**i**) composite soft magnetic material shell (X); (**j**) composite soft magnetic material shell (Y); (**k**) iron shell (X); (**l**) iron shell (Y).

Initially, the series device was tested: two coils were connected in series, and the total resistance of the coils was measured to be 5.2 Ω using a multimeter. The oscilloscope channel 1 was connected to the ends of the series coil. The spring position was adjusted to different scales (in 5 mm increments, from 0–100 mm), as illustrated in Figure A4a. Resonant frequency identification was conducted by sweeping excitation frequency while monitoring output voltage and oscillator amplitude. The natural frequency was determined when frequency deviation from peak response induced these parameters’ attenuation. Figure 9 illustrates the experimentally measured tuning range of the natural frequency of the series device, which is 4.5 Hz to 16.7 Hz. The tuning range extends up to 12.2 Hz, exhibiting satisfactory linearity of tuning. In the low frequency range below 4.5 Hz, manufacturing errors in the device and the choice of housing material resulted in a pendulum with a small amplitude, which did not allow effective collection of vibration energy. Subsequently, the power generation performance of the tandem unit was evaluated through a series of tests, and the soft magnetic shell made from different materials was compared. When the natural frequency of the device is 16.7 Hz and 10 Hz, respectively, the excitation is input from the XY direction, and the value of the induced electromotive force generated is recorded. The results are presented in Table 3 and Table 4, and the oscilloscope counts are shown in Figure A5. The series coil resistance was measured as 5.2 Ω using a multimeter, and the power was calculated. The EVEHD demonstrates the capacity to produce considerable outputs in a multitude of directions of input excitation, exhibiting slight variations in the results. These include a maximum output voltage of 1.92 V and 708.9 mW of power in the X-direction, and a maximum output voltage of 2 V and 769.2 mW of power in the Y-direction.

**Figure A6:**
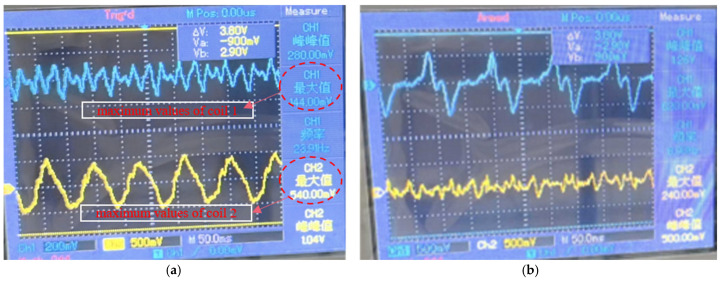
Experimental results of device in parallel mode. (**a**) Input 9.3 Hz excitation, X-direction; (**b**) input 13.3 Hz excitation, Y-direction.

Subsequently, experiments were conducted on a parallel device, and two distinct coils linked to oscilloscope channels 1 and 2, respectively. The range of natural frequencies of the parallel device was measured in a manner similar to that employed for the previous experiment, as illustrated in Figure 9. The range of natural frequencies that can be tuned by spring 1 is 3.7 Hz–9.3 Hz, while the range tunable by spring 2 is 5 Hz–13.3 Hz. The position of springs 1 and 2 was adjusted so that the natural frequency of the device is 9.3 Hz and 13.3 Hz, respectively. The capture of low-frequency vibration energy was improved by selecting more suitable housing materials for parallel devices. When an excitation with a frequency of 9.3 Hz in the X-direction is input, the waveform of the induced voltage in coil 1 is clearly noticeable, while coil 2 exhibits only a trace voltage. When the Y-direction frequency is 13.3 Hz, the value of the induced voltage in coil 2 is more pronounced, and there is only a trace voltage in coil 1. The two coil resistances were ascertained to be 2.5 Ω and 2.7 Ω, respectively. Table 5 illustrates that the maximum induced voltage in coil 1 is 540 mV and the power is 116.6 mW, while the maximum induced voltage in coil 2 is 620 mV and the power is 142.4 mW (the oscilloscope counts are shown in Figure A6). The parallel configuration achieves multidirectional/multifrequency energy harvesting, though its power output and voltage generation underperform compared to the series configuration. The output of the multidirectional EVEH is connected to the energy harvesting circuit, and the voltage at the output of the energy harvesting circuit is found to be stable at 3.24 V.

The power conversion efficiency (η) is quantified as:

(28) $$\eta =\frac{P_{in}}{P_{out}}\times 100\%$$

where $P_{out}$ is the output electrical power, and $P_{in}$ is the input mechanical power calculated from excitation force F and velocity v:

(29) $$P_{in}=F\times v=F\times (2\pi fA)$$

Laboratory measurements of A are approximately 3 mm. Substituting the data, the efficiency of the series mode is 46.1% and 53% at frequencies of 10 and 16.7 Hz, respectively. The efficiency of the parallel mode is 29.9% and 33.1% at frequencies of 9.3 and 13.3 Hz, respectively. These values demonstrate competitive performance of this multidirectional EVEHD. There are some differences between the actual efficiency and the ideal efficiency due to manufacturing errors such as manual winding of coils and measurement errors during testing.

## 5. Conclusions

This study demonstrates a dual-mass EVEHD with tunable frequency (4.5–16.7 Hz in series mode; dual-band 3.7–9.3/5–13.3 Hz in parallel mode) through spring-position modulation, achieving multidirectional energy capture and X/Y-axis excitations yielding a maximum of 1.92 V/708.9 mW and 2 V/769.2 mW for series mode and parallel mode, respectively. Parametric optimization of magnet–coil–soft magnetic assemblies enhanced power density by 49.9 and 85.2%, while dual-configuration versatility enables either voltage amplification (series) or multifrequency harvesting (parallel). The energy management circuit can generate a 3.24 V DC output. Experimental validation confirms the device’s adaptability to complex vibration environments, with future work expected to target adaptive spring positioning and omnidirectional harvesting capabilities.

## Figures and Tables

**Table 1:** Main parameters of the model.

Project	Value
The length of the pendulum, $L_{1},L_{2}$	110 mm
Front end quality, m	0.09 kg
Pendulum quality, $m_{1}$	0.03362 kg
Vertical distance from the center of rotation, $a_{1},a_{2}$	10–100 mm
Elastic coefficient, $k_{1},k_{2}$	1326.718744 N/m

**Table 2:** Initial parameters of static simulation.

Project	Numerical Value
Radius of PM	7.5 mm
Number of PMs	8
Length of PM	3 mm
Inner radius of coil	14 mm
Outer radius of coil	16 mm
Width of air gap	6.5 mm
Length of shell	60 mm
Inner radius of shell	16 mm
Outer radius of shell	17 mm

**Table 3:** Performance of the series device with input excitation along the X-direction.

Natural Frequency	Without Soft Magnetic Shell		Composite Soft Magnetic Material Shell		Iron Shell	
	Voltage/V	Power/mw	Voltage/V	Power/mw	Voltage/V	Power/mw
16.7 Hz	1.42	387.8	1.36	355.7	1.92	708.9
10 Hz	1.08	224.3	1.1	232.7	1.22	286.2

**Table 4:** Performance of the series device with input excitation along the Y-direction.

Natural Frequency	Without Soft Magnetic Shell		Composite Soft Magnetic Material Shell		Iron Shell	
	Voltage/V	Power/mw	Voltage/V	Power/mw	Voltage/V	Power/mw
16.7 Hz	1.46	409.9	1.52	444.3	2	769.2
10 Hz	1.24	295.7	1.3	325	1.66	529.9

**Table 5:** Performance of the parallel device with input excitation along different directions.

Frequency	Direction of Excitation	Coil 1		Coil 2	
		Power/mw	Voltage/V	Power/mw	Voltage/V
9.3 Hz	X	116.6	0.54	11.5	0.176
13.3 Hz	Y	23	0.24	142.4	0.62

## Data Availability

The data are available from the corresponding author on reasonable request.

## Appendix A. Derivation of Motion Equations for System

In the case of horizontal excitation, the potential energy V, kinetic energy T, and dissipation energy D corresponding to the dual-mass pendulum model in series are, respectively:

(A1) $$V=V_{E}+V_{G}\;$$

(A2) $$V_{E}=\frac{1}{2}\sin^{2}\left(\theta +\theta_{0}\right)\left(k_{1}a_{1}^{2}+k_{2}a_{2}^{2}\right)\;$$

(A3) $$V_{G}=-mgL_{1}\sin(\theta +\theta_{0})+mgL_{1}\sin\theta_{0}-mg\;[L_{2}-L_{2}\cos\left(\theta +\theta_{0}\right)]+mg(L_{2}-L_{2}\cos\theta_{0})$$

(A4) $$T=T_{m}+T_{r}\;$$

(A5) $$T_{m}=\frac{1}{2}m\left\{{{\;[\dot{x}}_{g}+\dot{\theta }L_{2}\cos(\theta +\theta_{0})]}^{2}+{\;[\dot{\theta }L_{2}\sin\left(\theta +\theta_{0})\right]}^{2}\right\}+\frac{1}{2}m\left\{{{\;[\dot{x}}_{g}-\dot{\theta }L_{1}\sin\left(\theta +\theta_{0}\right)]}^{2}+{\;[\dot{\theta }L_{1}\cos\left(\theta +\theta_{0})\right]}^{2}\right\}\;$$

(A6) $$T_{r}=m_{1}{{\dot{x}}_{g}}^{2}-\frac{1}{2}m_{1}\dot{\theta }L_{1}{\dot{x}}_{g}\sin\left(\theta +\theta_{0}\right)+\frac{1}{2}m_{1}\dot{\theta }L_{2}{\dot{x}}_{g}\cos\left(\theta +\theta_{0}\right)+\frac{1}{6}m_{1}{\dot{\theta }}^{2}{(L}_{1}^{2}+L_{2}^{2})\;$$

(A7) $$D=\frac{1}{2}\left(c_{t}+c_{p}\right)\left(L_{1}^{2}+L_{2}^{2}\right){\dot{\theta }}^{2}$$

where θ represents the rotation angle of the pendulum, $x_{g}$ is the base displacement, $L_{1}$ and $L_{2}$ are the lengths of pendulums, and $a_{1}$ and $a_{2}$ are the distances of the two springs from the center of rotation. m is the mass of the block; $m_{1}$ is the mass of the pendulum. $c_{t}$ and $c_{p}$ are the electromagnetic damping and parasitic damping coefficients, respectively; $V_{E}$ and $V_{G}$ represent the elastic and gravitational potential energy of the system, respectively; $T_{m}$ and $T_{r}$ represent the kinetic energy of the mass block and the pendulum, respectively. Assuming that the rotation angle of the pendulum is small, the trigonometric function of Eq can be simplified. According to the Lagrange method, the corresponding equation of motion is:

(A8) $$\frac{\partial }{\partial t}\left(\frac{\partial L}{\partial \dot{\theta }}\right)-\frac{\partial L}{\partial \theta }+\frac{\partial D}{\partial \dot{\theta }}=0\;$$

where L=T−V, and the result can be obtained as follows:

(A9) $$\begin{array}{c} \left[\left(mL_{2}+m_{1}L_{2}\right)\cos\left(\theta +\theta_{0}\right)-\left(mL_{1}+m_{1}L_{1}\right)\sin\left(\theta +\theta_{0}\right)\right]{\ddot{x}}_{g}+\left(L_{1}^{2}+L_{2}^{2}\right)\left(m+\frac{1}{3}m_{1}\right)\ddot{\theta }+\\ \left(c_{t}+c_{p}\right)\left(L_{1}^{2}+L_{2}^{2}\right)\dot{\theta }-\left(mgL_{2}-k_{1}a_{1}^{2}-k_{2}a_{2}^{2}\right)\theta -\left(mgL_{2}-k_{1}a_{1}^{2}-k_{2}a_{2}^{2}\right)\theta_{0}-mgL_{1}=0 \end{array}$$

The derivation of the parallel dual-mass pendulum has been omitted.

## Appendix B. Simulation Results of Coils of Different Specifications

**Table A1:** Induced voltage and power in the coil with different wire specifications.

**Outer Diameter of Wire/mm**	Radius of Coil $r_{0}$/mm	R/Ω($K_{coil}=0.9$)	U/V	Power/mw
0.19	14.93	12.72	1.6587	216.3
0.2	14.95	11.29	1.6406	238.4
0.21	14.97	10.02	1.6751	280
0.225	15	8.51	1.6266	310.9
0.235	15.02	7.64	1.6013	335.6
0.255	15.06	6.3	1.6512	432.8
0.275	15.1	5.28	1.6395	509.1
0.35	15.6	2.92	1.6269	906.4
0.39	15.72	2.24	1.5528	1076.4
0.45	15.9	1.59	1.5402	1492
0.56	16.79	0.91	1.5238	2551.6
